# Impact of Maternal Essential Fatty Acid Intake on the Birth Weight of Infants

**DOI:** 10.34763/jmotherandchild.20232701.d-22-00052

**Published:** 2023-10-16

**Authors:** Manojna Masina, Srujana Medithi, Arti Muley

**Affiliations:** Symbiosis Institute of Health Sciences (SIHS), Symbiosis International (Deemed University), Pune, Maharashtra, India

**Keywords:** Essential fatty acids, infant birth weight, maternal nutrition, omega-3 fatty acids, omega-6 fatty acids, birth outcomes

## Abstract

**Background:**

Increased uptake of essential fatty acids during pregnancy through seafood and supplementation has been shown to positively correlate with gestational age and increased infant birth weight. We aimed to evaluate the effect of maternal dietary intake of essential fatty acids, supplementation on gestational period and infant birth weight.

**Materials:**

A literature search with the help of various databases such as PubMed, Google Scholar, Web of Science and Scopus was conducted.

**Methods:**

Original research articles and intervention-based studies, which involve an association between dietary intake and supplementation of essential fatty acids during full-term pregnancy on human infant birth outcomes and published from 2011 to 2021, were included.

**Results:**

In total, there were 21 intervention-based studies, including full-term pregnant women with or without existing comorbidities, which compared essential fatty acids in the form of dietary sources and supplementation with dietary counseling and with or without placebo. The intervention trials included in this review were conducted in developed and developing countries. Half of the pregnant women who enrolled in the study had comorbidities such as diabetes and hypertension, which might increase their risk of adverse maternal and infant birth outcomes. Most of the studies included in the review have reported a positive association between improvised dietary and supplementation intake of essential fatty acids with increased length of gestation, infant birth weight and other parameters such as head circumference, infant birth length and growth velocity.

**Conclusion:**

Positive correlations were found between increased consumption of essential fatty acids in food sources and supplements with improvised infant birth weight and gestational period.

## Introduction

Birth weight is a crucial predictor of the health trajectory of an infant. Inadequate food intake or lack of a balanced diet during pregnancy can cause a birth weight of less than 2.5 kg, indicating low birth weight (LBW) and preterm birth. Preterm delivery is the primary cause of infant death and is linked to a higher risk of several detrimental health effects in childhood and adulthood [1]. Even in well-nourished populations, various dietary factors and the quality of nutritional patterns affect the course and outcome of pregnancy [2]. The nutritional status of pregnant women, especially during the first and last trimester, is a crucial determinant for maintaining a healthy infant birth weight. The current nutritional recommendations for pregnant women emphasize a personalised approach in nutritional counselling, considering women's accessibility to food, ethnicity, race, food choices and body mass index [3].

Every cell in our body requires essential fatty acids, which serve as precursors of eicosanoids and are physiologically active chemicals that help in cell division and proliferation. Docosahexaenoic acid (DHA), primarily present in oily fish, is essential for early human development because it enhances visual, intellectual and kinesthetic development [4]. Foods high in essential fatty acids are crucial for the growth of the embryonic neurological and immune systems [5]. Essential polyunsaturated fatty acids (PUFA), abundant in fish oil, seafood, nuts and oilseeds, are necessary for the growth and maturation of various organ systems in our body, particularly the brain and eyes, throughout fetal development. Higher maternal omega-3 to omega-6 PUFA ratio is linked to improved fetal health, fetal growth velocity, increased birth weight and extended gestation periods [2]. A minimum of 300 mg of eicosanoid acid and 200 mg of DHA should be consumed by pregnant women to meet the increased requirements to carry out various organ functions [6].

Additionally, consuming seafood, which is high in essential fatty acids, may boost birth weight in two ways: by increasing the ratio of physiologically active prostacyclins to thromboxanes, lowering the viscosity of blood, and also by aiding in the rapid growth of the fetus and prolonging the length of the gestation period [7]. A transplacental gradient in favour of the fetus has caused an increase in the percentage of PUFA in the fetal circulation, primarily from the mother's blood in the second trimester of pregnancy, which accompanies the most significant growth spurt and is probably sufficient to support rapid cell division. Although adipose tissue makes up only 14% of birth weight, total fat mass accounts for roughly 50% of birth weight fluctuation. Compared to neonates of average weight, LBW children were found to have reduced omega-3 and omega-6 PUFA status [2]. Maternal supplementation of DHA is essential since these levels are associated with fetal DHA levels that reflect increased DHA storage to support nursing with minimal changes in pregnancy duration, early baby growth and cognitive development [8]. Pregnant women who have consumed supplementation of 600 mg of DHA per day had an elevated red blood cell (RBC) count and DHA content. Preterm and LBW babies would be less frequent if gestation periods were longer and larger birth sizes. Supplementation of fish oil during pregnancy resulted in a longer gestational length and an increased birth weight due to higher intrauterine growth. It also emphasizes the dose-response relationship between the amount of long-chain polyunsaturated fatty acids (LCPUFA) supplement intake and its effect on gestational length and fetal growth with a minimal threshold [9]. Women under the gestational age of 34 weeks were given a higher daily dosage of supplementation with 1000 mg of DHA than 200 mg. They were found to experience a declined early preterm birth rate and increased birth weight [10]. In this review, we have focussed on the impact of maternal essential fatty acid intake on the birth weight of infants.

## Material and Methods

### Search strategy

An intervention-based Cochrane review was conducted using the databases PubMed, Google Scholar, Web of Science and Scopus. The Cochrane handbook for systematic reviews of interventions was used as the guide (2008). In the present review, articles that were published in the English language between the time period 2011 and 2021 were selected. The keywords used in the literature search were ‘essential fatty acids and infant birth weight’, ‘maternal fatty acid intake’, ‘maternal nutrition’, ‘pregnancy and DHA’, ‘gestational length’, ‘omega-3 fatty acids’, ‘omega-6 fatty acids’, ‘fish oil supplementation’, ‘arachidonic acid’, ‘essential fatty acids’, ‘prolonged gestation’, ‘fetal growth’, ‘birth outcomes’, ‘gestational age’, ‘nutrition’ and ‘essential fatty acids’. Boolean expressions indicating ‘OR’ and ‘AND’ were used while completing the literature search. The generated lists of publications were manually reviewed to identify studies that addressed the objectives of the present investigation and to classify the papers according to their associations with the consumption of essential fatty acids during pregnancy and childbirth weight. The present review follows PRISMA guidelines, which are usually used in systematic reviews that evaluate healthcare interventions and present review involves mixed method with qualitative and quantitative research methods. For relevant information for some items, such as dietary interventions and supplementation of essential fatty acids which have already appeared in a publicly accessible review protocol, referring to the protocol was deemed sufficient. Alternatively, detailed descriptions of the methods used, or additional results, were reported. Selection of papers was based on considering all the study characteristics used to determine a study's eligibility for inclusion in the review, that is, components described in the PICO framework or one of its variants, and other characteristics, such as eligible study design for example dietary interventions of essential fatty acids during pregnancy, setting of the intervention and minimum duration of follow-up period, were considered.

### Study identification and selection

The published reports mentioning the study population as full-term gestation period in pregnant women aged 17 to 40 years with or without existing comorbidities were included in the present review. The study population includes participants from all over the world. Original research articles that correlate intake from food sources and supplementation of essential fatty acids during full-term pregnancy with infant birth outcomes in human studies were considered. The uptake of essential fatty acids during pregnancy can influence the composition of breast milk, which eventually affects infant development. Hence, research articles stating the related mechanisms were also included. The eligibility and intervention-based studies and placebo and active control interventions with actual intervention group studies were also considered. Studies were excluded if they were animal studies, systematic reviews and an overview of reviews based on exclusion criteria. The title of each chosen published report was carefully checked to avoid duplication of articles.

Furthermore, the abstracts from the selected research articles were also thoroughly read and evaluated to determine their relevance to our topic. The articles were chosen based on their abstracts. Then the full text of the articles was examined. This thorough scrutiny checks for eligibility included 21 intervention-based articles in this review.

### Data retrieval

The following data were obtained from the selected published reports: stage of pregnancy, existing comorbidities, place/country of the study, sample size, age of the participant (years), dietary sources of EFA, type and dosage of the supplement, supplementation strategy, association with gestational length and infant birth weight.

The main aim of this review article is to determine the associations between dietary intake and supplementation of essential fatty acids during full-term pregnancy and infant birth weight, gestational length and other parameters such as infant birth length, head and neck circumference, and cognitive development. The articles were divided into the following sub-domains to help understand how essential fatty acid consumed during pregnancy relates to infant birth weight:

1. The relationship between maternal dietary essential fatty acid intake with infant birth and gestational duration.
2. The effect of essential fatty acid supplementation during pregnancy on the birth weight of infants.
3. The impact of dietary consumption of essential fatty acids and their supplementation during pregnancy influence newborn birth weight.

## Results

Searches in databases included 66,900 references. The literature search of Scopus (1309), Pubmed (2357), Google Scholar (56,440) and Web of Science (6740) from 2011 to 2021 and additional 21 records from other sources were retrieved. From the reference list, 500 records were screened according to the inclusion and exclusion criteria, out of which 40 records were excluded, and 21 studies were included (Fig 1). Out of 21 selected research articles, there were 11 randomised controlled trials, 3 observational studies, 5 cohort studies, 1 clinical report and 1 cross-sectional study. Table 1 describes various factors representing the stage of pregnancy, any existing comorbidities, and the participant's age. Among the studies listed, one randomised trial and cross-sectional, three observational and cohort studies were shown to positively correlate dietary intake of essential fatty acids in vegetable oils, nuts and seafood with prolonged gestational length and increased birth weight [11, 12, 13, 14, 15, 16, 17]. In contrast, one observational study reported a negative association between LBW and increased consumption of dietary sources of essential fatty acids [18]. Other parameters were also reported, such as increased head circumference [13, 15] and infant length [15].

**Table 1.** Studies indicating the consumption of maternal dietary essential fatty acid and the observed infant birth and gestational duration

**Stage of pregnancy**	**Existing comorbiditis**	**Place/country of the study**	**Number of women enrolled**	**Age of the participant (years)**	**Dietary sources of EFA**	**Association with gestational length (Y/N)**	**Infant birth weight (Y/N)**	**Other parameters**	**Reference**
First trimester	None reported	Bangalore, India	1838	17–40	Total fats, short-chain fatty acids, ALA, LC n-3 PUFA intake (fish, fats/oil, eggs, cereals, nuts, milk, and vegetables)	NA	Mixed results (inverted U shaped) High and low birth weight was seen	Gestational age	14
16^th^–20^th^ week of gestation (singleton)	None reported	Department of Obstetrics and Gynecology, Bharati hospital Pune, India	111	NA	Omega-3 and 6 fatty acids, SFA, MUFA, AA, DHA	NA	DHA-increased birth weight LCPUFA-low birth weight	NA	16
Third trimester	None reported	Torbat-e Jam, Mashhad, Iran	453	18–40	Total fat intake	NA	Yes, positively associated	Head circumference and length of the infant	13
33–41 weeks	Diabetes, hypertension, chronic diseases	Isfahan, Iran.	225	NA	Foods high in protein, such as bread and cereals, fruits, vegetables, shrimp, and fish	Yes, positively associated	Yes, positively associated	NA	11
16^th^–20^th^ week of gestation	No medical or obstetrical complications.	Department of Obstetrics and Gynecology, Bharati hospital Pune, India	NA	NA	Food sources rich in omega-3 fatty acids were noted using food frequency questionnaire	No.	DHA-positively associated with birth weight	Maternal total erythrocyte omega-6 fatty acid and infant length	15
8–28 weeks	None reported	United Kingdom	1289	18–45	SFA, MUFA, and PUFA	Yes, increased gestational length up to 40 weeks	Yes, negatively associated with lower birth weight	Birth centile	18
<17 wk of gestation	None reported	Turku and neighboring areas in south-west Finland	256	NA	Dietary counselling	Yes, increased gestational length up to 40 weeks	Yes, positively associated	NA	17
>32 week of gestation (data unpublished)	None reported	NA	282	19–40	Tempeh, tofu, and the water extracts of some legumes like mung bean and soybean	Yes, low intake of ALA with lower gestational weight, DHA, and EPA were not associated	Yes, low intake of ALA with lower birth weight and DHA and EPA has not been associated	NA	12

**Abbreviations:** ALA - Alpha-linolenic acid, AA- Arachidonic acid, DHA- Docosahexaenoic acid, EPA - Eicasopentanoic acid, LC n-3 PUFA - Long-chain omega-3 poly-unsaturated fatty acids, MUFA- Monounsaturated fatty acids, NA – Not Available, SFA-short-chain fatty acids, PUFA -Polyunsaturated fatty acids

Table 2 shows various demographic factors representing the stage of pregnancy, any existing comorbidities and age of the participant. This table particularly emphasizes the importance of supplementation of essential fatty acids with various factors indicating the type of supplement, supplementation strategy and the supplement dosage given to pregnant women. Six of the seven studies, including five randomised trials and one cohort study, have reported a beneficial association with increased infant size at birth. The supplementations provided consisted of algae-derived DHA, omega-3 fatty acids, and fish oil capsules with increased supplementation of above 600 mg and daily consumption throughout the pregnancy [19, 20, 21, 8, 22, 9], except one of the randomised controlled trials, which did not report any associations with infant birth weight and other parameters; instead, increased gestational length was a secondary outcome [23]. Other parameters include birth length, head circumference, total essential fatty acid level [20], decreased morbidity [19], higher nutrition education, supplemental uptake [21] and high DHA status [8].

**Table 2:** Studies indicating the consumption of essential fatty acid supplementation during pregnancy and the observed birth weight of infants.

**Stage of pregnancy**	**Place/country of the study**	**Existing comorbidities**	**Number of women enrolled**	**Age of the participant**	**Type of supplement**	**Dosage of supplement**	**Supplementation strategy**	**Association with gestational length (Y/N)**	**Infant birth weight (Y/N)**	**Other parameters**	**References**
≤ 20 weeks	India	None reported.	1200	18–35	Algal DHA supplement	635 mg soft gel capsules	Started between 12 and 18 weeks of pregnancy, with a median value of 15 weeks	No, as a secondary outcome	No	Length and head circumference at the time of birth	23
8–20 weeks of gestation	Kansas city, USA	Diabetes mellitus, high blood pressure, and other chronic diseases.	350	16–36	Marine algae-oil source of DHA	Three capsules/day	In the placebo group, three capsules containing 50/50 soybean and maize oil containing 200 mg of DHA were given	Yes, gestational length was found to be high	Yes, infant birth weight was the high	Birth length, head circumference, and total fatty acid in blood	20
First trimester – singleton pregnancy	India	N None reported	200	25 years	Omega-3 fatty acid capsules	MEGA3 contains eicosapentatonic acid (EPA) 180mg + docosahexaenoic acid (DHA) 120mg/day	(MEGA3) Supplementation was started in the first trimester at the first visit and iron and calcium supplement to other group followed till 28 weeks	No	Yes, increasing birth weight	Morbidity rate	19
12 and before 20 weeks gestation	Kansas City metropolitan area	NA	NA	20–30	DHA and corn and soybean mixture	600 mg of DHA per day or a placebo (corn and soybean combination)	Intake of supplementation after 12 weeks of pregnancy till the end of gestation period	NA	Yes, decreased birth weight	Maternal age	21
16–20 of pregnancy	Tabriz, Iran	NO	150	18 to 35	Fish oil capsule and placebo	Daily supplementation of fish oil capsules containing 1000 mg and placebo intervention of capsules with 1000 mg of liquid paraffin	One capsule was taken each day with a total of 140 capsules taken till the end of intervention	NA	Yes, increasing birth weight but is not statistically significant	Head circumference and length of the infant	22
24 wks of pregnancy	From prospective Danish study	Diseased conditions	736	NA	Fish oil capsule and olive oil	1g of fish oil capsule containing (55% of EPA and 37% DHA and other intervention group with 1 g of olive oil containing (72% of oleic acid and 12% omega-6 linoleic acid)	Daily supplementation of one capsule per starting from 22 week till delivery	Yes, prolongation of gestational length and age	Yes, increase in birth weight	NA	9
20–36 weeks of gestation	Department of Obstetrics and Gynecology of IIMCT-Railway Hospital Rawalpindi	No	500	32	Omega-3 fatty acid supplementation	NA	Omega-3 fatty acid supplementation was given to group A participants from a gestational period and participants in the Group B have not received any such treatment	Yes	Yes	Fetal DHA stores	8

**Abbreviations:** DHA- Docosahexaenoic acid, EPA - Eicasopentanoic acid, NA – Not Available

Table 3 shows various demographic factors representing the stage of pregnancy, any existing comorbidities and the participant's age. This table evaluates essential fatty acid supplementation and food sources and their association with gestational length and infant birth outcomes, such as infant birth weight, head circumference and essential fatty acid concentrations in maternal and fetal plasma. All of these studies were conducted outside of India with a participant age group of more than 18 years. Five randomised controlled trials reported a favourable association between increased gestational length and infant birth weight [24, 25, 26, 27, 28]. One clinical report reporting positive associations with infant birth weight but showed no association with gestational length [29]. Other parameters were also included indicating increased dietary intake and supplementation with enhanced birth outcomes [24], significantly increased birth weight, birth length [29, 26], fetal fat mass, growth [25] and omega-3 and 6 fatty acids ratio [27].

**Table 3.** Studies indicating the dietary consumption of essential fatty acids along with supplementation strategy during pregnancy and their observed influence on newborn birth weight.

**Stage of pregnancy**	**Place/country of the study**	**Existing comorbidities (Y/N)**	**Number of women enrolled**	**Age of the participant (years)**	**Dietary sources of EFA**	**Type of supplement**	**Dosage of supplement**	**Supplementation strategy**	**Association with gestational length (Y/N)**	**Infant birth weight (Y/N)**	**Other parameters**	**References**
12 to 28 weeks of gestation	Korea	NA	1407	30	Saturated, monounsaturated, and polyunsaturated FA	Dietary supplement	NA	NA	No.	Yes, Increase in birth weight	Length of the newborn	29
<16 weeks)	Metro Health Medical Center/Case Western Reserve University, Ohio	Obesity/increased 3:n6 ratio	72	NA	Dietary sources of omega-3 and omega-6 PUFA and saturated fatty acids	Omega-3, DHA, EPA	Two capsules twice a day	Oral supplementation of 800 mg containing docosahexaenoic acid (DHA) and 1200 mg of eicosapentaenoic acid(EPA) altogether making around 2000 mg of n-3 PUFA until delivery	Yes, increased gestational length with supplementation	Yes, increased birth weight with supplementation	Fetal fat-free mass and fetal growth	25
≥34 completed weeks of gestation	Royal Prince Alfred Hospital, Sydney, Australia	NA	224	30–35	Total n–3 PUFA and the individual PUFAs, ALA, EPA, DHA, and LA.	Fish oil supplementation	NA	NA	No	ALA-yes	NA	28
16- and 22-weeks’ gestation	13 academic medical clinics in the United States	Obesity and lean body weight	852	NA	Fish intake	Oral capsule of DHA and n3 fatty acids	placebo group with (inert mineral oil) or 2 g of n3 FA and intervention group with a daily consumption consisting a combination of 800 mg of docosohexaenoic acid with 1200 mg of eicosapentaenoic acid (EPA)	Daily supplementation throughput the pregnancy	Yes, shorter gestational length	Yes, increased birth weight	Maternal plasma n6: n3 FA ratio and fetal growth	27
>15 weeks of gestation	Munich area, Germany	No	208	18–43	Reduced ratio of n−6 to n−3 LCPUFAs from 7:1 and moderate intake of arachidonic acid, abundant in meat products and eggs	Fish-oil supplement	Marinol D-40; Lipid Nutrition containing 1200 mg n−3 LCPUFAs (1020 mg DHA and 180 mg EPA)	Daily supplementation throughout pregnancy	Yes, showed prolonged gestational length but no significant difference with the control group (without supplementation and reduce intake only nutritional counselling being done)	Yes, showed increased birth weight but no significant difference with the control group	Head circumference and fat distribution	26
16 to 20 weeks of gestation	Colorado	No, healthy subjects	871	>18	Education arm with increased intake with 300 mg of docosahexaenoic acid from fish and other dietary sources	Algal derived docosahexaenoic acid (or) olive oil placebo	300 to 600 mg of algal derived docosahexaenoic acid (or) olive oil placebo	DHA was provided in the form of supplement bars containing docosahexaenoic acid CsOS oil with energy of 300kcal. Gel capsules containing olive oil were given to those who refused to consume nutrient bars, and upplementation was initiated at week 20 of gestation and continued until delivery	Yes, showed a prolonged gestational length of 4 days.	Yes, showed increased birth weight	Maternal DHA levels, fetal DHA status and cognitive development	24

**Abbreviations:** ALA - Alpha-linolenic acid, DHA- Docosahexaenoic acid, EPA - Eicasopentanoic acid, FA – fatty acid, LA- Linoleic Acid, LC n-3 PUFA - Long-chain omega-3 poly-unsaturated fatty acids, NA – Not Available, OWOB- Overweight over obese

## Discussion

### Relationship between maternal dietary essential fatty acid intake with infant birth and gestational duration

DHA has been found to help maintain the fluidity of membrane channels, regulate ion channels to enable synaptic transmission and serve as a substrate for binding to membrane receptors, all of which help to promote neurodevelopment [30]. As there is a decreased concentration of essential fatty acids in maternal plasma, especially the DHA during the first and second trimesters, the requirements increase with the rapid development of the neural and retina [25]. The fetal adipose tissue is used to store DHA. After birth, this fatty acid is swiftly transported into blood circulation to ensure optimal growth and development throughout the crucial neonatal phase. The fetal supply of fatty acids is influenced by maternal nutritional intake, placental transfer and release of fatty acids from the mother's adipose tissue [2]. A positive correlation was found between increasing seafood consumption during pregnancy and newborn birth weight and head circumference. Lean fish consumption was identified as the critical factor in this connection. Women who consumed more than 60 g of seafood had significantly lower rates of reduced birth weight than those who consumed a diet with little or no seafood intake [31].

Several intervention-based studies suggest a relation between maternal essential fatty acid intake, gestational length and infant birth outcomes. Omega-3 fatty acids have been shown to improve the lipid bilayer membrane's fluidity and receptor-induced activity. Essential fatty acids could boost the growth of the fetus by increasing placental blood flow and lowering blood viscosity with the help of omega-3 fatty acid metabolites like prostacyclin and thromboxane. The study by Mani et al., [32] highlights the importance of short-chain fatty acids intake in carbohydrate-rich maternal diets, as well as the relative impact of omega-6 and omega-3 fatty acid intake, which initiates progress in the rapid growth that occurs between 32 and 38 weeks of the gestational period as the fetus's weight nearly doubles. Another study has observed that DHA levels in maternal erythrocytes were positively related to birth weight, implying that DHA may have a role in determining birth weight. Dietary adaptation for optimal maternal fatty acid status aids in promoting fetal growth, which may help improve the child's health status later in life and have ramifications for the offspring's neurodevelopment.

Additionally, a correlation between dietary fat intake during pregnancy and infant newborn weight highlights the importance of essential fatty acids in the growth and development of the fetus. This correlation shows that for every gram of fat consumed, the infant's mean weight, height, and head circumference increase by 3.92 g, 0.013 cm and 0.008 cm, respectively [13]. Likewise, if the median total intake was lower than the recommended dietary allowance of 0.82 g/dl, it was associated with decreased gestational age and lower birth weight [12]. Several studies have reported that pregnant mothers who have consumed different kinds of seafood such as fish and shrimp with at least 5 g in a week have more extended gestation periods and increased infant's birth weight [11]. According to a prospective study by Meher et al. [16], DHA levels were positively associated with infant weight in the first trimester of the gestational period, which highlighted the importance of LCPUFA in developing LBW. The arachidonic acid (AA) levels in maternal plasma, granulocytes, and omega-6 fatty acids found in the blood circulation were higher in the LBW group throughout the second trimester with a statistical significance of (*p* < 0.05). The findings of Niinivirta et al. [17] emphasized the relevance of maternal diet for child health, implying that pregnant women should receive dietary counselling. Pregnant women who received dietary guidance had a better supply of necessary fatty acids, which is especially crucial during the period of rapid development. Conversely, a study found an unfavourable connotation between total fat uptake during pregnancy and its subcomponents, such as PUFA, linked to reduced birth weight and birth centile [18].

Most of the studies have reported positive associations with increased essential fatty acid intake and increased infant birth weight as these studies have considered dietary interventions, which includes essential fatty acid-rich sources in common. Women enrolled for these interventions have not reported any existing comorbidities, and few papers have shown positive relations with increased gestational length. One of the studies showed negative association, as the with an additional intake of 10 g of total fat intake was associated with lower birth weight; however, specific types of fat ingested was not considered.

### Effect of essential fatty acid supplementation during pregnancy on the birth weight of infants

Studies on the effects of prenatal supplements, particularly larger dosages of omega-3 fatty acids of up to 600–800 mg/day, have shown promising benefits in improving gestational age and better childbirth outcomes. Increased birth weight was seen in the Western population due to increased uptake of omega-3 fatty acids supplements. In a randomised controlled study, a double-blinded placebo control group, 400 mg of algal supplementation was given throughout the pregnancy. The primary outcome obtained was associated with increased birth weight, birth length, and head circumference. The secondary outcome obtained was an increased length of gestation [23]. A similar randomised clinical trial that was carried out in which pregnant women with pre-existing comorbidities were supplemented with a marine algae source of DHA of 600 mg per day, and the results obtained were found to have positive associations with the length of the gestational period and increased infant birth size, head circumference and the total amount of fatty acid levels in maternal plasma [20]. A notable observation was seen with an increased weight of the infant with an average gain of 320.74 g. Still, no association was found with the duration of gestation, since 89% of the study population had achieved normal delivery [19]. A study by Ostadrahimi et al. [22] analysed that a reduced dosage of fish oil supplementation helps boost neonatal birth weight, but the difference was not statistically significant. The intervention group with fish oil supplementation had a much lower incidence of LBW. Supplementation with fish oil capsules during the third trimester of pregnancy had a considerably bigger gestational age, which shows that the rise in birth weight is majorly due to higher intrauterine growth and the longest pregnancy period [9]. The results showed that fish oil intake prolongs pregnancy by 2 to 4 days and increases birth weight by 70 to 170 g. Omega-3 fatty acid oral supplementation during high-risk pregnancies significantly increased the gestational period and birth weight. Omega-3 fatty acid levels are significantly transferred trans-placentally, affecting both the mother and the fetus. Similar findings were reported in another study that showed a difference in mean gestational age between the omega-3 supplemented and non-supplemented groups with a standard deviation of 38.2 and 36.6, which was statistically significant at the time of birth [8]. Maternal uptake of DHA supplements dramatically increases the gestational period and infant birth size in high-risk pregnancies. Maternal DHA levels are associated with fetal DHA levels, which implies greater DHA storage to support the baby's nursing with a modest increase in gestational length, early baby growth and skill development. Furthermore, by altering the equilibrium of markers of prostaglandins indicating PGE2 and PGF2 as well as prostacyclin, omega-3 fatty acids help reduce the likelihood of consequent preterm delivery and decreased weight of the infant at the time of birth. The hepatic production of omega-6 fatty acids, which serve as antecedents to PGE2 and PGF2 alpha, declines in response to increased omega-3 fatty acid intake. Conversely, a clinical trial revealed that DHA at a dose of 600 mg daily throughout the second half of pregnancy prevented early preterm birth at 34 weeks of gestation and LBW of 1500 g [21].

Several studies, where interventions were randomised controlled trials, which included sources either from marine algae or fish oil capsules that contained substantial high amounts of essential fatty acids ranging from 10% to 70% of total fat consumed in the form of a capsule regularly throughout gestation period, have shown increased birth weight and lower incidences of preterm birth. One of the studies did not show any association, as the supplementation was introduced in the second trimester, and the source of supplement was purely from DHA excluding other essential fatty acids. Multiple factors such as intervention throughout the pregnancy, family support, stress levels and gestational length were not considered.

### Dietary consumption of essential fatty acids and their supplementation during pregnancy influence newborn birth weight

Besides the dietary sources of essential fatty acids, supplementation is essential in increasing an infant's birth weight, psychomotor skills and gestational age. Dietary supplementation is essential when a person finds it challenging to meet prescribed macronutrient intake through diet alone or dislikes a particular food. Preterm and LBW infants are usually born with neurodevelopmental problems due to a lack or inadequate supply of essential fatty acids in the diet of pregnant women; conversely, supplementation of omega-3 and 6 fatty acids is required for maintaining the optimal fatty acid status of the mother and newborn infant [25]. Based on various studies on postnatal dietary interventions, it was observed that supplementation of essential fatty acids, especially DHA supplementation, has improved the neuronal developmental status and the infant's birth weight. Fish oil supplementation to the pregnant women showed significant increase in fetal DHA and neonatal DHA status [10].

Increased intake of essential fatty acids helps extend the gestational age and weight of the infant at birth by lowering the chances of LBW infants and helps maintain the flow of fatty acids in the blood circulation [29]. Triacylglycerol and cholesteryl ester AA concentrations in cord plasma were positively correlated with gestational age, birth weight and length [11]. Increased uptake of fats obtained from marine sources such as fish and whales were correlated with a minimal extension of gestation period consecutively with a 1% relative increase of phospholipids DHA content in maternal cord serum, with a reduced birth weight adjusted for gestational age [28]. Fish oil capsules consist of essential fatty acids, such as EPA and DHA, which are associated with a longer gestation duration. This may be because it alters the ratio of prostaglandins derived from AA [32]. However, some studies have reported negative associations with the consumption of essential fatty acids, which have been found to lower birth weight but showed positive associations with carbohydrates and their components with increased birth length and birth centile [18].

The investigation by Lee et al. [29] stated that the growth of the fetus depends on the supply of essential fatty acids during pregnancy that is essential and deficiencies of any of these essential fatty acids could have unfavourable consequences on the birth size and height of the infant at the time of birth, and length of gestational period. Dietary intake of essential fatty acids such as DHA and AA are crucial for a baby's growth throughout the pregnancy. An inverted U-shaped relationship was observed between maternal essential fatty acid intake and birth weight that may be seen in the group of pregnant women with lower intake of essential fatty acids and are associated with lower birth weight. Furthermore, women who adopted a Western diet during pregnancy, rich in omega-6 fatty acids and a dietary supplement, had higher rates of small gestational age (SGA), implying that omega-6 fatty acid intake may also influence SGA baby birth rates among Western people. Another study concluded that when participants were given daily supplementation of 2 g of DHA along with an intake of foods rich in omega-3 and omega-6 essential PUFA starting from early pregnancy with a total duration of 14 weeks until the end of gestation, infants were born with a week longer gestational age and an increased birth weight of 343 g and 0.44 standard deviation unit [25]. Surprisingly, the increased fetal growth was not due to increased adiposity but rather to increased fat-free mass accumulation. A randomised controlled trial by Penfield-Cyr et al. [27] was a secondary analysis conducted among overweight and obese pregnant women with an improvised ratio of omega-6 fatty acid to omega-3 fatty acid during mid-gestation, which was linked to lower fetal growth and shorter gestation length. However small and analytically unrelated, these associations may provide the framework for future studies in high-risk populations. With implications for fetal growth and health in pregnant women, the ratio of omega-6 to omega-3 fatty acids may be a critical metabolic indication. Impairment of embryonic development has been associated with increased inflammatory burden, notably maternal plasma ratio of omega-6 to omega-3 fatty acids.

Furthermore, higher omega-3 fatty acids and omega-6 fatty acids were linked to shortened gestation length in over obese pregnant women. Phang et al. [28] investigated the food frequency questionnaire and plasma lipid profile of pregnant women to show that mothers with lower birth weight infants with a higher maternal alpha-linoleic acid intake of about 221 g than those with the lowest intake. Much et al. [26] emphasized that the concentrations of DHA and EPA in the umbilical cord blood were found to have a strong negative relationship with parameters indicating newborn fat mass at the time of birth. However, at the 32nd week of the gestational period, the concentrations were beneficial to the infant's birth length and gestational age with reduced consumption of omega-3 to omega-6 LCPUFAs from 7:1 and a moderate intake of AA, abundant in meat products and eggs. Another study evidenced nutrition education about significantly improving the dosage of DHA by about 300 mg per day obtained through food sources such as fish and other nutritional sources in the form of food bars and fish oil supplements [24]. Results were positively associated with DHA-derived food sources with a greater gestational length for four days and birth weight, especially in low-income groups with a low risk for preterm birth.

One of the major parameters to be considered is the initiation of dietary intervention and the supplementation during pregnancy, which have been reported in previous studies, wherein initiation in first and third trimester is crucial for birth weight of the infant. The regions where seafoods are less consumed, which are rich in essential fatty acids, are more prone to lower birth weight, reported especially in Indian studies. The lowest tertile of EPA intake and risk of LBW in the third trimester showed a similar significant connection. These patterns were also present in the first trimester, and throughout all trimesters, there was no appreciable difference in the relationship's slopes between birth weight and logarithmically transformed fish intake. Gestational weight was not given much importance in previous observational studies; however, increased gestational length has been shown to be associated with increased birth weight and with significant intake of essential fatty acids along with supplementation throughout the pregnancy, and quality and dosage plays a major role in development of fetal growth.

## Conclusion and Future Directions

It is widely known that unfavourable conditions during pregnancy will lead to malformation in fetal development and can lead to abnormal birth weight and preterm birth. This review emphasizes studies reporting positive correlations between maternal fatty acid uptake, improvised birth weight and gestational length. However, the mechanisms underlying the importance of these predetermined consequences are still poorly understood. Some studies have reported a negative correlation between increased uptake of essential fatty acids and decreased infant birth weight. This may be because the studies were carried out during certain selected periods throughout the pregnancy. More intervention-based studies involving nutrition education should emphasize the need for essential fatty acid uptake during pregnancy. At the same time, some of the studies with longer follow-up periods have reported no association with birth weight as nutrient values were calculated by the software for sub-components based on the database linking the composition of food items, which might not be precise or comprehensive, and a combination of dietary assessments with reduced bias can be made while conducting further studies. Therefore, to further establish the ideal intake of essential fatty acids and their components for long-term maternal and infant birth outcomes, it is crucial to take into account both the absolute content of essential fatty acids, the dosage of its components and the ratio of omega-6 fatty acids to omega-3 fatty acids in experimental investigations.
